# Supplementary material for: Can short PROMs support valid factor-based sub-scores? Example of COMQ-12 in chronic otitis media
Source: PLoS One. 2022 Sep 29;17(9):e0274513. doi: 10.1371/journal.pone.0274513 (PMC9522295; doi:10.1371/journal.pone.0274513)
Supplement: S1 Appendix — (DOCX) [file pone.0274513.s001.docx]

Supplementary Information - S1 Appendix

Can short PROMs support valid factor-based sub-scores? Example of COMQ-12 in chronic otitis media

Bojana Bukurov^1,2*^, Mark Haggard^3^, Helen Spencer^4^, Nenad Arsovic^1,2^, Sandra Sipetic Grujicic^1,5^

1. Faculty of Medicine, University of Belgrade, Serbia
2. Clinic for Otorhinolaryngology and Maxillofacial Surgery, University Clinical Centre of Serbia, Belgrade, Serbia
3. Department of Psychology, University of Cambridge, UK
4. Independent member, Eurotitis Study group
5. Institute for Epidemiology, Belgrade, Serbia

*Corresponding author:

Bojana Bukurov

Email: [bojana.bukurov@med.bg.ac.rs](mailto:bojana.bukurov@med.bg.ac.rs), [boianabukurov@gmail.com](mailto:boianabukurov@gmail.com)

Incorporation of replicate data from Phase 1 study and the use of dual-visit baseline

For the previously published data on the first 60 cases who gave baseline replicate data on COMQ-12 at 2^nd^ visit (V2, 4 weeks after the first) we required decision between two options: simply using visit 1 (V1) data only or pre-averaging over the two values. The slightly greater complexity for the latter is overridden by the scientific obligations to maximise precision and to use all relevant data held. The issue does not arise for SF-36, which was not acquired at Visit 2. The derivation to create factor score formulae using CFA (Table 2) had to be done on V1 data only, because structural equation modelling cannot readily handle such irregular data structures, but elsewhere the available data for the 60 cases with two baseline visits were averaged for reliability.

As to detail, we tested for consequently increased leptokurtosis from such averaged inclusion of part-replicate data but did not find it at statistical significance. The V1-V2 difference in 1^st^ PC total for COMQ-12 on the 60 cases having both is the most sensitive measure of equivalence, and this was well below 0.10 of 1 SD. The gross difference made by improving data quantity in only approximately a quarter of cases (60/246) is thus small but averaging the two baseline scores slightly reduces measurement error for those 60 cases while honouring the stated inclusion principle. This evident high representativeness of the V2 attenders is probably due partly to the homogeneous clinic caseload over time, and partly to absence of major determining factors in accepting a 2^nd^ pre-operative visit. In the 2nd-iteration EFA (3-factor Varimax on all 246 cases with scaled items), the percentage of item variance explained was 58.52 (V1 averaged with V2 data where present) versus 58.16% (V1 only), small but in the expected direction so there was no reason to override the principle of maximum use of relevant data acquired. Cross-tabulations of the dual-baseline 60 versus the single-baseline 186, gave no significant differences between cases with V2 and cases without V2, in any of the clinical and demographic variables, even for audiometry -- the most precise of the baseline measures.
