# Supplementary material for: Can short PROMs support valid factor-based sub-scores? Example of COMQ-12 in chronic otitis media
Source: PLoS One. 2022 Sep 29;17(9):e0274513. doi: 10.1371/journal.pone.0274513 (PMC9522295; doi:10.1371/journal.pone.0274513)
Supplement: S2 Appendix — (DOCX) [file pone.0274513.s002.docx]

**Supplementary Information – S2 Appendix**

**Can short PROMs support valid factor-based sub-scores? Example of COMQ-12 in chronic otitis media**

Bojana Bukurov^1,2*^, Mark Haggard^3^, Helen Spencer^4^, Nenad Arsovic^1,2^, Sandra Sipetic Grujicic^1,5^

1. Faculty of Medicine, University of Belgrade, Serbia
2. Clinic for Otorhinolaryngology and Maxillofacial Surgery, University Clinical Centre of Serbia, Belgrade
3. Department of Psychology, University of Cambridge, UK
4. Independent member, Eurotitis Study Group
5. Institute for Epidemiology, University of Belgrade, Serbia

*Corresponding author:

Bojana Bukurov

Email: [bojana.bukurov@med.bg.ac.rs](mailto:bojana.bukurov@med.bg.ac.rs), [boianabukurov@gmail.com](mailto:boianabukurov@gmail.com)

**Scoring and handling of missing data**

*Relation to Data File in S5*

At the level of the minimal dataset in the file in S5 the major source of missingness is highly visible as it is designed- in, as set out in Appendix S1. That replication of the COMQ-12 data on 60 of the cases was previously published, and the remaining 186 are ‘missing’ in only one sense and marked as such by ‘system missing’ in the SPSS data file (.). They are not subject to biased attrition in the way that other forms of missing data, entered as ‘-999’ prior to any simple or complex imputation, might be. The details are specified in the main text and supplementary appendices 2 and 4, also reflected, to assist tracing, in certain variables' labels. This contributes to making some of the labels very long because they contain the definition and explanation eg clarification of derivation history. In replication attempts the consequently inelegant layout is probably worth keeping to help distinguish versions of variables that might become confused; but if this becomes bothersome, use of the name only obviates the problem (except in graphics where the length reappears.)

The dataset supplied goes beyond the totally minimalist approach, by providing certain variables that could also be derived anew from other data also given, leading to 149 variables in all, more than the minimum expected from the main text and tables. This extra provision is restricted to the main path of variables shown to be useful in the analyses and used in Tables and for Conclusions, for example by excluding 2-factor solutions. To have not provided some of this filling in (and so left the copious derivation work that we undertook as a kind of enabling technology for the substantive results) would have excessively discouraged replication of Results.

The very few instances of missing items due to failure to respond, or to procedural mishap at acquisition might make the aggregate complexity of the set of imputation procedures seem disproportionate (as details cannot make a great difference to results if few cases are involved) , but for individual missing types we have tried to keep complexity and effort proportionate. The occurrence of imputation is seen in the Data File when a missing value at an earlier stage of derivation becomes filled at a later one. The brevity of allusions to missing data in the main text is justified by: (a) the gross low missing rates at baseline visit; (b) the lack of specific need in the analyses of baseline data to adjust for attrition bias; and (c) the present Supplementary Information. The procedures were set out in the Phase 1 publication, but their application formulae are revised here in the light of a fourfold increase in sample size and consequent slightly changed percentages missing [1]. Imputation is applied because, from both a bias and power/sensitivity point of view, and limiting both Type 1 and Type 2 errors, it is almost always better to impute than not to [2].

*COMQ-12 missing data*

Only 4 item-cases were missing at all at baseline, i.e., 4 patients with a single missing item each (items 2, 3, 5, 10). The overall probability of an individual item being missing is 4/(12 x 246)=0.001355. The 60 2^nd^ visit patients in the subsample documenting reliability had no missing data at all, so the following basic procedure was applied to V1 data only, before the averaging of V1 and V2 used for most variables (preceding S1 Appendix).

Aiding equal-interval measurement and imputation by item scaling

For COMQ-12, scaling involved using as the preliminary continuous dependent variables for each of the item scaling regressions, the respective factor scores from the first iteration on the basic numerical rating data. Here 'respective' refers to the factor on which the item under consideration loads most highly. This alignment is clear with one exception, the cross-loading item 12. This was therefore scaled against the first principal component (PC), not a factor score. The preliminary dependent variables for scaling were defined by the initial 3-factor Varimax EFA (for the 12 items with numerical ratings and using preliminarily imputed means of items for missings at V1). The parameter estimates for each item as categorical independent variable when predicting this factor score in the first iteration were adopted as the scaled values, and a scaled value was also assigned to 'missing' responses by floating the category and estimating this 'missing' category's value in the regression. Scaled values were used in a 2^nd^ iteration EFA where the items now all had scaled response levels, to provide final weighting for the scoring formulae. For this we implemented the item scaled values from the first iteration in place of the raw values.

It is important to note that scaling regressions do not explicitly standardise or transform for normal residuals, and this applies also to the model which linearises the relationship of binaural audiometry data to the’ hearing’ factor score. This is because to transform would subvert the scaling; also, the several response-level category estimates in effect superimpose their error distributions around them, already making the overall scaling model residuals slightly closer to normal. However extreme skew as with SF-36 does come through into scaled versions.

The fact that factor scores are automatically standardised imposes a set of re-weightings – in one sense now standardised – potentially different from that taken by the unscaled items in the first iteration. For example, an item of which the scaling has particularly increased the correlation with other related items included in the preliminary total for the same measure) will see an increase in its weighting. This scaling could be done externally with a regression, for example here making a binaurally weighted audiometric measure the dependent variable in scaling regressions; however, regression against an external variable is not advised for determining item scaled values, as in regression, the items redundant upon highly correlated stronger items are drastically down-weighted, to the detriment of reliability. For this reason and because in real datasets there may exist no such external validity constraint, as there is not for two of our sub-score factors, we use the internal consistency constraints of factor analysis, on which the weighting is given by factor-loading. For the present dataset, we scaled by internal consistency, linearising against a preliminary factor score. This is more laborious, replacing one stage by two. However, the investment in this is justified; it offers a scoring system for later visits, or indeed for any other comparable set of COMQ-12 data (eg having a smaller sample than the present) or other circumstance which could make, using the present scoring formulae preferable to those from a new small-sample derivation). Unless the scaled values are imposed from a single scaling exercise non-stationarity of measurement may occur, eg between visits, even if the content-based labelling of separate factor solutions suggests high similarity of factor structures. Non-stationary measurement error can be considerable where loadings shift, as they can.

In the scaling, there was only one ordinality violation out of 5 by 12 = 60 adjacent pairings in the scaled estimates for the 12 items; it occurred on item 5 ('discomfort around the ear'); here the two response-categories involved were collapsed into one for reliability and the regression re-run, so both original categories received the same final scaled value, trading precision for reliability. After these stages of scaling and re-running of the factor analyses and PC total score, the individual scaled item response values and the scores for each patient were saved for subsequent use, including in the CFA. The resulting scaled values are visible in the summary data file submitted as S5 Appendix. This item scaling process is one variant upon item-response theory principles; this appendix does not alter the specification of it given in the prior publication [1] but confirms applicability and optimises scale values and weights on the 4 times larger dataset.

Only slight advantage for scaling was shown previously in the sample size of 60, in terms of strength of factor solution. Claiming only near equivalence, we continued chiefly to use the method for scaling items described there, but now on 246 cases, chiefly with three additional reasons to undertake scaling; (a) the regressions for scaling include rational imputation for missing items, very similar to maximum likelihood imputation in character; (b) because linearity and equality of intervals are thereby addressed; and (c) category collapsing of sparsely populated categories before imputation of missing data provides more reliable estimation of an appropriate scaled value, although pooled.

Furthermore, the larger sample size might have produced a differing measure of the advantage, and once the tests have been run the application require little further effort. The correlations given in the next paragraph show general consistency between item scoring methods on the full sample but only small advantage for correlations from item scaling, compared to taking the numerical rating responses at face value, as the raw data are already numerical ratings not verbal categories.

On Varimax EFA 3-F as overall best structure, we next compared the three item scoring approaches in the same terms obtaining: for scaled, solution Rsq 0.582, (with third, ie last, rotated eigenvalue [LREV] 1.70); for raw numerical rating Rsq 0.568 (LREV 1.67); for nearest-median dichotomised Rsq 0.512 (LREV 1.87). The inter-scoring-method correlations bore out this small advantage for item scaling. For numerical rating data, unscaled seems non-inferior toscaled for the correlations summarised in factor analysis, but it was important to establish this for reasons more fully stated below. Pairwise correlations in order of factors extracted: F1, F3, F3 were: scaled with numerical r=0.994, 0.994, and 0.991; scaled with dichotomous r = 0.916, 0.821, 0.684; and numerical with dichotomous r=0.909, 0.817, and 0.726. This pattern defines the scaled and the numerical rating as comparably better than dichotomous. The higher correlation particularly for F3 values between scaled and numerical item scoring follows the factor analysis model fit (as Rsq) in suggesting that good quantification can strengthen precision for weaker factors, partly off-setting paucity of high-loading items. The negligibly small disadvantage for the numerical rating means that its equal-interval properties now been adequately established for purposes of totaling, correlation, and factor analysis, which had not previously been done. The 6-level ratings are thus non-degrading, as well as pragmatically attractive, relative to the effort of further item scaling using them. We do not claim that the scaling advantage seen with verbal quantifiers as data source is seen to the same extent on numerical ratings and there are reasons why it would not be [3]. However, linearity and equal-interval scales are crucial for quantifying correlation strengths, so we used the scaled versions, in which investment had already been made. Testing equal-interval properties has gone halfway to implementing scaling for this study and shown that this need not be repeated in application.

*Distributions of 1^st^ PC total and other factor-based scores*

The distributions of factor-based variables emerging from imputation and CFA (as used in the main correlations Table 3) are given on the left side of the table below. They are not far from normal and on inspection were found to be sufficiently linearly interrelated to not need transformation to strengthen inter-relationships. (When weighting audiometry binaurally, see S3, we transformed the binaural predictor to linearise with the questionnaire score but not the variables in the prediction regression itself.) In the same table's final two columns are distribution characteristics for the adopted auditory criterion measures for 'hearing' factors, namely the audiometrically predicted values for the ‘hearing’ factor. Technically, these are the predicted values from bivariate regressions, of the respective 'hearing' scores as dependent variables, using the available monaural aPTA values, as independent variables. The transformation of the predicted value for the ‘hearing’ score on COMQ-12 (from the threshold values) was next applied as natural logarithm with no constant added to the audiometrically predicted values predicting bi-factor 'hearing', and the constant 0.866 subtracted for the formula predicting simple CFA 'hearing'. Some of the indices of distribution shape exceed two SEs; however, this is a fairly large sample and the values are not extreme. The subsequent Pearson correlations require good interval measurement, not normality of distribution, and for the aPTA regressions this consideration is balanced with seeking linearity to legitimately maximise correlations, done comparably for the two solutions but also visibly making each nearer to normal when transformed than when not.

**Table S2. Skewness and kurtosis of COMQ-12 total and sub-scores with two CFA structures; also, the predicted values from linear regressions to weight the auditory thresholds in predicting 'hearing' scores.**

|  | | | | | | Raw  ‘Hearing’  predicted  values | Transformed  Hearing’  predicted  values |
| --- | --- | --- | --- | --- | --- | --- | --- |
| Solution | Distribution | 1^st^ PC | Hearing | Activities/ Healthcare | Ear discharge |  |  |
| SimpleCFA | Skewness  Kurtosis | 0.209  -0.659 | 0.252  -0.880 | 0.078  -1.209 | -0.021  -1.165 | 0.752  0.737 | -0.595  0.488 |
| Bi-factor | Skewness  Kurtosis | 0.372  -0.605 | -0.097  0.204 | 0.151  -1.040 | -0.012  -0.775 | 0.823  0.870 | 0.020  -0.321 |

Table footnotes. Note that throughout, pre-transformed SE values for N=246 are: SE (skewness) = 0.155, SE (kurtosis) = 0.309. The generally flat-topped (negative kurtosis) properties of the factor score descriptives are notable as a general property of the instrument, slightly lessened in the bi-factor solution.

The distributions for the predicted values from binaural hearing weighting regressions have a different form and the purpose in showing their effect on predicted values differs; for these it is appropriateness and comparability in the weighting regressions. With 'hearing' untransformed, in raw form both regressions give positive skew and kurtosis. Slightly differing binaural weightings are obtained between the two factor solutions as specified in the S3 Appendix. All non-normality indices are reduced for model predicted values in the last column by the transformation of the predicted values mainly for linearity. However, despite the intention to enable fair comparison, perfect control is impossible: the remaining difference obtained between the two solutions in the skew and kurtosis of ‘hearing’ residuals, in the prediction from audiometry, reflects the fact that the best (bi-factor) solution greatly changes the 'hearing' factor sub-score by extracting into the general factor that item variation not reflecting hearing disability.

*SF-36 missing data*

Only 14 item-cases were missing. These were distributed as follows: two patients each had item 27 & two separate ones had item 28 missing. The remaining 10 instances involved patients with a single missing item (2, 6, 9, 20, 21, 22, 25, 30, 31, & 35). The overall probability of an individual item being missing is thus 14/(246 x 36) i.e., 0.001581, very similar to that for COMQ-12. Despite the slight preponderance of lower-down items missing, there is as expected from the designed-in brevity, no convincing evidence here for length of instrument as such being a burden and determinant of rate of missing items.

The imputed values for these missing SF-36 data were obtained as follows. For non-scalable (i.e., originally dichotomous) items with only one degree of freedom, no data were missing. For scalable items we followed the 2- pass procedure as described for COMQ-12, except that we used 1^st^ PC of all 36 items as the most reliable and simple weighted total for preliminary dependent variable in the scaling regressions, and not factor scores, as these were not required for the generic application in this article. The 1^st^ PC is strong (43.9% of total item variance for the scaled version) and the detailed factor structure weak (with much cross-loading, reflected by the cumulative item variance explained with 3 Varimax factors, rising only to 56.01% of total). This strong 1^st^ PC supports empirically for SF-36 the a priori decision to use a weighted total, exemplified by 1st PC. We therefore based imputation on the regression category estimates (as done for COMQ-12) using the 1^st^ PC as reliable preliminary total, for which the few missing items comprise only a small part of the available information. Likewise, this was followed by a 2^nd^ iteration using the scaled values. Distribution and linearity issues for the SF-36 1^st^ PC are addressed in the main text as preliminary to correlations, and further comments on factor structure plus explanation of the versions included in the data file S5 are given in Appendix S4.

**References for S2 Appendix**

1. Bukurov, B., Arsovic, N., Grujicic, S.S., Haggard, M., Spencer, H., Marinkovic, J.E. (2017). Psychometric characteristics of the chronic Otitis media questionnaire 12 (COMQ -12): stability of factor structure and replicability shown by the Serbian version. Health Qual Life Outcomes, 15(1):207.
2. van der Heijden, G.J., Donders, A.R., Stijnen, T., Moons, K.G. (2006). Imputation of missing values is superior to complete case analysis and the missing-indicator method in multivariable diagnostic research: a clinical example. J Clin Epidemiol, 59(10):1102-9.
3. Milovanovic, J., Filipovic, S.A., Marchisio, P., Haggard, M.P., Zhang, M.F., Spencer, H. (2016). Eurotitis-2 Study Group. Precision-scored parental report questions and HL-scaled tympanometry as informative measures of hearing in otitis media 1: Large-sample evidence on determinants and complementarity to pure-tone audiometry. Int J Pediatr Otorhinolaryngol, 83:113-31. doi: 10.1016/j.ijporl.2016.01.
