# Supplementary material for: Can short PROMs support valid factor-based sub-scores? Example of COMQ-12 in chronic otitis media
Source: PLoS One. 2022 Sep 29;17(9):e0274513. doi: 10.1371/journal.pone.0274513 (PMC9522295; doi:10.1371/journal.pone.0274513)
Supplement: S3 Appendix — (DOCX) [file pone.0274513.s003.docx]

Supplementary Information – S3 Appendix

Can short PROMs support valid factor-based sub-scores? Example of COMQ-12 in chronic otitis media

Bojana Bukurov^1,2*^, Mark Haggard^3^, Helen Spencer^4^, Nenad Arsovic^1,2^, Sandra Sipetic Grujicic^1,5^

1. Faculty of Medicine, University of Belgrade, Serbia
2. Clinic for Otorhinolaryngology and Maxillofacial Surgery, University Clinical Centre of Serbia, Belgrade
3. Department of Psychology, University of Cambridge, UK
4. Independent member, Eurotitis Study group
5. Institute for Epidemiology, Belgrade, Serbia

*Corresponding author:

Bojana Bukurov

Email: [bojana.bukurov@med.bg.ac.rs](mailto:bojana.bukurov@med.bg.ac.rs), [boianabukurov@gmail.com](mailto:boianabukurov@gmail.com)

Definition of an appropriate binaural average hearing level (aPTA) for asymmetric hearing loss

The purpose of this definition is to avoid two serious errors that would result from a simplistic convention-based indicator choice for the criterion measure with which the COMQ-12 'hearing' factor score is to be correlated in assessing criterion validity. Using only the better ear (ie in almost every case the 'unaffected ear' from the point of view of COM and recommendation for surgery) would provide a commonly used, though incomplete, definition related to auditory disability. However, this discards information, specifically about moderate to severe hearing impairments on the worse ears, which in this sample have wide enough range to contribute importantly to the variation in any credible better/worse ear weighting scheme. The variation in the better ear is narrower, as well as the mean being lower; hence, in relation to error of measurement, the better-ear-only approach would inevitably produce low correlations and so would underestimate criterion validity. Giving equal binaural weighting is a valid simplification for populations with largely symmetric hearing losses; however, whilst reducing the clamping effect of the narrower range of better-ear values, this would not remove the compression of variance completely. The sample size of 246 is large enough that the scientific advantage shifts from adoption of an a priori conventional or externally data-derived formulation, to estimation of optimum internal to the data set and so best 'tuned' to the properties of the specific population.

It is necessary here to overcome a reflex rejection of circularity as being un-informative, and to leave behind historical emphases on non-nullity ('significance' of some unspecified difference or association). That is irrelevant here; it has long been known that there nearly always does exist an audiometry with 'hearing' relationship for any credible sample size, that between measured sensitivity and a reported hearing score. The point is rather that this relationship is often weak due to other influences than sensitivity, and therefore in fairness to the present 'Hearing' factor, the appropriate audiometric index has to be optimised by derivation from the particular data including the questionnaire score of which the criterion validity is being assessed. The table below shows a generalisation ('multi-versing') across the three types of factor solution mentioned in the main text. Whilst there are slight differences between coefficient ratios across the various factor solutions, sufficient similarity emerges to justify proceeding.

**S3 Table. Regressions to establish most predictive weighting of binaural hearing thresholds for predicting ‘Hearing’ factor scores from COMQ-12 questions**

| Model | Coeff | SE | t | P | ή_p_^2^ |
| --- | --- | --- | --- | --- | --- |
| 'Hearing' Varimax EFA 3-F (Adj Rsq=0.054) | | | | | |
| aPTA affected ear | 0.008 | 0.003 | 2.384 | 0.018 | 0.023 |
| aPTA other ear | 0.008 | 0.004 | 2.254 | 0.025 | 0.020 |
| 'Hearing' CFA 3-F (Adj Rsq=0.070) | | | | |  |
| aPTA affected ear | 0.007 | 0.002 | 2.783 | 0.006 | 0.031 |
| aPTA other ear | 0.006 | 0.003 | 2.436 | 0.016 | 0.024 |
| 'Hearing' Bi-factor 3-F (Adj Rsq=0.050) | | | | | |
| aPTA affected ear | 0.003 | 0.001 | 2.002 | 0.046 | 0.016 |
| aPTA other ear | 0.004 | 0.001 | 2.459 | 0.015 | 0.024 |

Table Footnotes. ή_p_^2^ -- partial eta-squared, the adjusted proportion of all variance in the dependent variable explained by this variable. The aPTA - pure tone averages of air-conduction thresholds - providing the independent variables, are calculated cross 4 relevant frequencies (0.5, 1, 2 and 4 kHz) on each ear. The difference between the simple CFA and bi-factor solutions in coefficient strength for the affected ear contribution is marginally statistically significant and we would have done this irrespective of whether either or both was significant. This is the appropriate because the purpose is to implement respective near optima for the two solutions in the main text Results Table 3, while not penalizing validity correlations for either. The main conclusion is not about one factor solution giving higher criterion validity than the other and the difference is explicable in terms of uncontrolled number of high-loading items; a possible small difference in ear dominance is beyond present scope. The lower two fields are used, further transformed, in main text Table 3, and the respective adjuster constants in the prediction equations are + 0.753 (simple CFA) and + 0.044 (bi-factor). Note that strictly the threshold data are from V1 only and that for reliability 60 of the cases average the questionnaire data over V1 and 2. S2 Appendix specifies the reason for this and for thus regarding the shift of mean timing in about a quarter of the cases as not material.

The coefficient values in the bottom pair of rows are those which maximise audiometric prediction for the bi- factor version of hearing and on the stated optimisation principle. The co-extraction of the non-specific variance in the bi-factor general factor of course weakens (compared to other solutions), the overall prediction of 'Hearing' questionnaire score from audiometry, as discussed in the main text, but the coefficients used (0.003, 0.004 and importantly the ratio between them) have been demonstrated to give optimal prediction, ie to maximise the correlation when this weighting is used in fairness to criterion validity.

This table also illustrates two properties of factor solutions supporting the analysis in the main text: (a) although conceptually similar, EFA Varimax and simple CFA on which that is based, do differ in their overall accuracy of prediction; (b) the generally found greater importance of the better ear for auditory disability re-appears in the bi- factor solution. Property (a) is due to discrete subsets of high-loading items, as in a subset's 1^st^ principal component or CFA, avoiding the statistical 'noise' created by inclusion in saved factor scores of low-loading items; this noise becomes amplified in assuring factor independence (orthogonality), an issue beyond present scope. Property (b) confirms that the larger better-ear coefficient shows the bi-factor solution as retrieving relatively more of the specific auditory disability, once the greater diverse symptomaticity (tinnitus, dizziness, etc) associated with the pathology has been extracted or at least reduced by extracting the general factor. That symptomaticity is what undermines the divergent validity of 'Hearing' factor scores and further argues for the separation of these variables, which are not separated unless a bi-factor solution is used. The details of the two ears' auditory sensitivity are not part of the HRQoL impact but without regard for them, the representation of that impact cannot be handled in the validation of the relevant PROM sub-score(s).
