# Supplementary material for: Can short PROMs support valid factor-based sub-scores? Example of COMQ-12 in chronic otitis media
Source: PLoS One. 2022 Sep 29;17(9):e0274513. doi: 10.1371/journal.pone.0274513 (PMC9522295; doi:10.1371/journal.pone.0274513)
Supplement: S4 Appendix — (DOCX) [file pone.0274513.s004.docx]

Supplementary Information – S4 Appendix

Can short PROMs support valid factor-based sub-scores? Example of COMQ-12 in chronic otitis media

Bojana Bukurov^1,2*^, Mark Haggard^3^, Helen Spencer^4^, Nenad Arsovic^1,2^, Sandra Sipetic Grujicic^1,5^

1. Faculty of Medicine, University of Belgrade
2. Clinic for Otorhinolaryngology and Maxillofacial Surgery, University Clinical Centre of Serbia, Belgrade
3. Department of Psychology, University of Cambridge, UK
4. Eurotitis Study group
5. Institute for Epidemiology, Belgrade, Serbia

*Corresponding author:

Bojana Bukurov

Email: [bojana.bukurov@med.bg.ac.rs](mailto:bojana.bukurov@med.bg.ac.rs), [boianabukurov@gmail.com](mailto:boianabukurov@gmail.com)

Composition of total SF-36 score for present purposes

Most publications using the SF-36 item set do very little to justify or specify the (scoring of the) variables used, although they may report their following precedents. Such standard formulation of measures is only one scientific goal. Neither of the two types of standard scoring (8 a priori content domains, two summary aspects, physical and mental) for SF-36 is highly adequate, and nor do they offer the needed single total with maximised aggregate reliability as required for our purpose of criterion validation. On the general principle of 5 items per construct (ie eventual factor) achieving adequacy, several of the 8-facet a priori content scores are under-sampled as to number of supporting items. Factor analyses of 36 items in SF-36 have been offered in the literature for extracting 2 to 5 and more scores; the sampling principle would permit use of 3 or 4 to be taken seriously, with use of any higher number to depend on details of factor solution offered in a large sample, and on the documented similarity of the sample in hand to the derivation sample. There is some attraction in a 3-F solution (for which factors can be labelled as physical limitations, activity restriction from those physical limitations, and 'mental' sub-score, and which we have used elsewhere.) The dominance of the 1^st^ principal component of variation entails that within supportable solutions the 3 factors must still inter-correlate highly in the present dataset (eg 0.44, 0. 39, 0.34) and in others, unless Varimax orthogonal rotation is used to imposed zero inter-factor correlation. This fact and the reliability requirement favour use here of the 1^st^ PC as weighted total of all items.

This foregoing short statement of fitness-for-purpose of the 1^st^ PC sits against the background of some possible differences in solution between cultures; a single generic total is less likely to show cultural differences than are differing interpreted factor solutions [1]. Also, not all published articles simply reporting an interpretable factor structure show sufficient detail of data to judge the statistical strategy and conclusions critically [2]. It is therefore hard to interpret existing results in a way conducive to favouring a particular number of factors or interpretative labels. Nevertheless, it is fair to note that psychometric difficulties about accepting the 2-domain summary (mental and physical) as a well-fitting structurally valid summary are not new [3] and therefore showing adequacy of the adopted solution for the application data set available is paramount.

The present Visit 1 data show a very strong 1^st^ principal component (after scaling, 1^st^ eigenvalue 15.79, with 43.9% of item variance explained) and only a weak factor structure, requiring a very strong argument for using anything other than this consistency-weighted total. The present article required a reliable generic total and did not need the 3-factor structure. This was to be used as criterion variable both for COMQ-12 total and with the 'activity limitations and healthcare uptake' sub-score, ‘activities/healthcare’ for short, to testify to their relevance and predictive power. Our data reduction to 1^st^ PC to achieve a single SF-36 aggregate therefore avoids consequences of the issue of non- independence of the 'mental' and 'physical' summaries discussed above; it also avoids multiple testing issues, where one factor might randomly appear to show a stronger correlation with COMQ-12 total than another, and so might attract undeserved attention or raise issues of interpretation. As the ‘physical problems’ listed in SF-36 items are related to pain and mobility, not to hearing and communication, there would probably no predictive validity advantage for the total over the 1^st^ factor with the 'mental' items, but here the importance of a priori aggregate reliability took precedence over possible specific interpretation and over minimising exposure to missing data, so we have preferred to use the weighted total.

Basis of the scoring formula used

The SF-36 data were suitable for factor analysis (with Kaiser-Meyer-Olkin measure of sampling adequacy = 0.942, and Bartlett Chi sq = 6344.448; DF=630; p<0.001). In EFA they gave 7 un-rotated eigenvalues over 1.0, but with only one sharp decline ('shoulder' in the scree plot), seen above the 1^st^ principal component, thus further directly justifying its use, because that reflects the strong general item, and hence factor inter-correlations (0.44, 0.39 and 0.34 in Simple CFA). This conveys the scientifically diminishing returns and poorly supported application rationale from extracting higher numbers of factors. The SF-36 items and their 1^st^ PC loadings are given in the table below. This defines the criterion measure adopted for the two validity appraisals. Crucially, the loadings as standardised regression coefficients are more generally meaningful than factor score coefficients, and so permit the replication of this study's results, on samples with differing variance and covariance. The negative skewness of the 1^st^ PC's distribution ('ceiling effect') in the absence of serious systemic disease, and the necessary transform to improve normality and linearity are addressed in the main text and Table 3 footnote. The 1^st^ PC loadings are homogeneously high across items (0.764 - 0.558) with the exception of the four lowest loaders, which invoke the wholly distinct issue of comparisons with other people, or with past and expected future. However, as these all load above 0.30 we have for reliability reasons not excluded these as might be done in prospective tailoring of a short form. Accordingly, this consistency-weighted 1^st^ PC can differ only very little in its correlations with other variables from some other (eg raw and unweighted) form of total. In practice, the differing item distributions and differing question formats (some dichotomous) would still require some form of pre-processing to truly achieve the standardised equal weighting implied by the idea of a 'raw' ie unweighted total: thus 'equal weighting' is the result of a process, not something inherent in the raw data. The ability to consider weighting and to impose reliability-based weighting via the principal component loadings, are here a by-product of the item scaling and subsequent 1^st^ PC procedure on standardised items as described in Appendix S2. The items are ordered in the table not in their numerical sequence but in fields according to their affiliations with the 3-factor Varimax solution, labelled as: 'non-physical' or mental; activity restriction-physical (including pain); and primary-physical. The often adjacent numberings in the order reflect that part of the patterned item inter-correlation giving factors for SF-36 is already favoured by the printed questionnaire format. Whilst does not permit dissociation of intrinsic meanings from contextual influences it favours an efficient instrument more than would random location.

*Interface to Data File S5 and explanation of range of values in scaled form*

The main text gives a summary of the issues in scaling the diverse item types in SF-36. Dichotomous items can only be scaled in the restricted sense of the final PC formula reweighting them relatively, in the additional stage for pre-scaled items. Given the few response levels and the non-numerical content, we considered it worthwhile to explore scaling the items with 3 or more and to use the scaled values where the standard error of the scaling estimates were low enough to believe that the change from the (falsely) equi-spaced coding clause (0,1,2 etc) was reliable -- in effect a significance criterion as reflecting the strength of evidence for some difference on this sample size of 246. In this process we also collapsed and fused cells with < 10 instances or violating ordinality. The information is potentially more widely useful for improved scoring of SF-36. The unscaled values are used for items (3,4,5,6,7,9,27) , the scaled ones used (1,2,7,10,11,12, 20-26, 38-35) where adopted, leaving the dichotomous unscalable ones (13-19). However, it has to be noted that 7, 10, 11 and 12 in effect became dichotomous after collapsing.st>. This division into item classes may appear inconvenient but it is not arbitrary, rather is evidence-based. The item number lists above assist reconciliation of the two forms of SF-36 data in appendix S5: rows 37-72, and 97-132 in ‘Variable view’ key and corresponding columns in ‘Data View’. The first set of given variables in the SPSS data file, in columns 37-72 are in the same sequence and with the same signing as in the paper questionnaire. Additionally, to assist reconciliation, the name element ‘highgood’ in the later block of scaled SF-36 variables reflects the fact that HRQoL measures generally score good quality of life as high positive value, so generating negative correlations with pathology-oriented measures; to simplify inspections at the item level, the exceptions to this generality have additionally been sign-inverted in this second set, which would not justify a largely redundant whole further column of data. This is why there are no negative loadings in the loadings table below. Similarly, the suffix ‘i’ as seen eg in "Q6SF36_highgood_V1i" and also items 9 and 27 reflects that having avoided the scaling stage generally used for a superior form of imputation, these items required an imputed value in the form of the sample mean, applying to the very few missing instances, respectively. This resulted in a single imputed mean value for items 6 and 9 but four values in all for 27, due to the sequence of processing stages and imputation.

Table with SF-36 items' 1^st^ PC total: item loadings for the score used as criterion measure

| **SF-36 item wording** | **1^st^ PC loading** |
| --- | --- |
| These questions are about how you feel and how things have been |  |
| with you **during the past 4 weeks**. For each question, please give the |  |
| one answer that comes closest to the way you have been feeling. |  |
| How much of the time during the **past 4 weeks**... |  |
| Q28. Have you felt downhearted and blue? | 0.724 |
| Q24. Have you been a very nervous person? | 0.670 |
| Q25. Have you felt so down in the dumps that nothing could cheer | 0.685 |
| you up? |  |
| Q26. Have you felt calm and peaceful? | 0.703 |
| Q30. Have you been a happy person? | 0.709 |
| Q31. Did you feel tired? | 0.723 |
| Q23. Did you feel full of pep? | 0.704 |
| Q29. Did you feel worn out? | 0.666 |
| Q20. During the **past 4 weeks**, to what extent has your physical | 0.743 |
| health or emotional problems interfered with your normal social |  |
| activities with family, friends, neighbours or groups? |  |
| These questions are about how you feel and how things have been |  |
| with you **during the past 4 weeks**. For each question, please give the |  |
| one answer that comes closest to the way you have been feeling. |  |
| How much of the time during the **past 4 weeks**... |  |
| Q27. Did you have a lot of energy? | 0.746 |
| Q32. During the **past 4 weeks**, how much of the time has **your** **physical** | 0.751 |
| **health or emotional problems** interfered with your social activities |  |
| (like visiting with friends, relatives etc)? |  |
| How TRUE or FALSE is **each** of the following statements for you. |  |
| Q36. My health is excellent | 0.637 |
| Q35. I expect my health to get worse | 0.464 |
| Q1. In general would you say your health is: Excellent/Very good/Good | 0.652 |
| Fair/Poor |  |
| How TRUE or FALSE is **each** of the following statements for you. |  |
| Q34. I am as healthy as anybody I know | 0.311 |
| Q2. **Compared to one year ago,** how would you rate your health in | 0.524 |
| general **now?** |  |
| During the **past 4 weeks**, have you had any of the following problems with |  |
| your work or other regular daily activities **as a result of your physical** |  |
| **health?** |  |
| Q15. Were limited in the **kind** of work or other activities | 0.679 |
| Q14. **Accomplished less** than you would like | 0.736 |
| Q13. Cut down the **amount of time** you spent on work or other activities | 0.720 |
| Q16. Had **difficulty** performing the work or other activities (for example, | 0.679 |
| it took extra effort) |  |
| Q22. During the **past 4 weeks**, how much did **pain** interfere with your | 0.764 |
| normal work (including both work outside the home and housework)? |  |
| Q21. How much **bodily** pain have you had during the **past 4 weeks** ? | 0.723 |
| The following items are about activities you might do during a typical |  |
| day. Does **your health now limit you** in these activities? If so, how much? |  |
| Q3. **Vigorous activities**, such as running, lifting heavy objects, | 0.693 |
| participating in strenuous sports |  |
| Q8. Bending, kneeling or stooping | 0.692 |
| During the **past 4 weeks**, have you had any of the following problems with |  |
| your work or other regular daily activities **as a result of any emotional** |  |
| **problems** (such as feeling depressed or anxious)? |  |
| Q17. Cut down the **amount of time** you spent on work or other activities | 0.714 |
| Q19. Didn't do work or other activities as **carefully** as usual | 0.617 |
| Q18. **Accomplished less** than you would like | 0.652 |
| The following items are about activities you might do during a typical |  |
| day. Does **your health now limit you** in these activities? If so, how much? |  |
| Q11. Walking **one block?** | 0.611 |
| Q10. Walking **several blocks?** | 0.653 |
| Q7. Climbing **one** flight of stairs**?** | 0.558 |
| Q9. Walking **more than a mile?** | 0.670 |
| Q12. Bathing or dressing yourself? | 0.599 |
| Q4. **Moderate activities,** such as moving a table, pushing a vacuum | 0.666 |
| cleaner, bowling, or playing golf? |  |
| Q5. Lifting or carrying groceries | 0.693 |
| Q6. Climbing **several** flights of stairs | 0.672 |
| How TRUE or FALSE is **each** of the following statements for you. |  |
| Q33. I seem to get sick a little easier than other people | 0.382 |
|  |  |

The Appendix References

1. Alhaji, M.M., Johan, N.H., Sharbini, S., Abdul Hamid, M.R., Khalil, M.A.M., Tan, J., Naing, L., Tuah, N.A.A. (2018). Psychometric Evaluation of the Brunei-Malay SF-36 version 2 Health Survey. Asian Pac J Cancer Prev, 19(7):1859-1865.
2. de Vet, H.C., Ader, H.J., Terwee, C.B., Pouwer, F. (2005). Are factor analytical techniques used appropriately in the validation of health status questionnaires? A systematic review on the quality of factor analysis of the SF-36. Qual Life Res, 14(5):1203-18.
3. Guthlin, C., Walach, H. (2007). MOS-SF 36: Structural Equation Modeling to Test the Construct Validity of the Second-Order Factor Structure. European Journal of Psychological Assessment, 23(1), 15-23.
